# Correlation Between the Online Visiting Time and Frequency Increase in Telemedicine Services Offered by Health Care Providers Before, During, and After the COVID-19 Pandemic in China: Cross-Sectional Study

**DOI:** 10.2196/65092

**Published:** 2025-02-26

**Authors:** Weiyi Wang, Yuntian Chu, Fangfang Cui, Xiaobing Shi, Xu Zhang, Dongxu Sun, Jinming Shi, Jie Zhao

**Affiliations:** 1 National Engineering Laboratory for Internet Medical Systems and Applications The First Affiliated Hospital of Zhengzhou University Zhengzhou China; 2 Institute of Intelligent Medicine Henan Academy of Innovations in Medical Science Zhengzhou China; 3 Shanghai Artificial Intelligence Laboratory Shanghai China

**Keywords:** telemedicine, post–COVID-19, provider’s perspective, length of online visit, COVID-19, pandemic, China, prevention, questionnaire, survey, healthcare provider

## Abstract

**Background:**

China has changed its COVID-19 prevention and control status since 2023. However, what role telemedicine will play post–COVID-19 is still uncertain.

**Objective:**

We aimed to determine the frequency change in health care providers offering telemedicine services before, during, and after COVID-19, as well as the correlation between the frequency change and telemedicine visit time.

**Methods:**

The Telemedicine Informationization Professional Committee of China (TIPC) carried out a nationwide questionnaire survey. We adopted data from part of the questionnaires that answered questions regarding the frequency of offering telemedicine services before, during, and after the COVID-19 explosion. Chi-square tests were applied to compare general differences in the between-group telemedicine frequency. Regression models were performed to analyze correlations between the frequency change and the time spent in online versus in-person visits.

**Results:**

Questionnaires from 428 providers were included. As reported, 39 (9.11%) providers often and 159 (37.15%) always offered telemedicine services before COVID-19 exploded. The component ratio increased to 12.38% (n=53) of providers often and 45.79% (n=196) always offering telemedicine during COVID-19 explosion and 12.62% (n=54) often and 50% (n=214) always offering telemedicine after pandemic control was relaxed. The increase in frequency shown as a difference between the before and during groups (χ2=17.21, *P*.002) and between the before and after groups (χ2=30.17, *P*<.001) was significant, while it was insignificant between the during and after groups (χ2=2.89, *P*.57). Senior professional titles (odds ratio [OR] 4.38, 95% CI 1.72-11.6) and longer (OR 3.87, 95% CI 1.95-7.89) and shorter (OR 2.04, 95% CI 1.11-3.87) online visits were correlated with the increase in frequency during versus before COVID-19. In addition, senior professional titles (OR 3.47, 95% CI 1.46-8.49), longer (OR 3.14, 95% CI 1.64-6.11) and shorter (OR=2.27, 95% CI 1.31-4.07) online visits, and using third-party telemedicine platforms (OR 0.51, 95% CI 0.29-0.86) were correlated with the increase in frequency after versus before COVID-19. No factor was significantly correlated with the frequency change after versus during COVID-19. In stratified analysis, longer online visits were correlated with both during versus before (OR 3.84, 95% CI 1.73-8.83) and after versus before (OR 3.40, 95% CI 1.61-7.34) groups for providers using hospital-run platforms, while shorter online visits were correlated with both during versus before (OR 8.16, 95% CI 1.39-68.3) and after versus before (OR 5.70, 95% CI 1.22-33.6) groups for providers using third-party platforms.

**Conclusions:**

The frequency of telemedicine has increased since the COVID-19 pandemic exploded and is correlated with the time spent in online versus in-person visits. The correlation is different for providers using hospital-run and third party platforms. On a hospital-run platform, providers with longer online visits have a higher frequency of offering telemedicine, while on a third-party platform, providers with shorter online visits are more likely to offer telemedicine.

## Introduction

The COVID-19 explosion has made significant changes to health care systems worldwide [1,2]. Telemedicine services, also referred to as virtual care in some studies, are defined in this paper as services that use digital technologies to provide health care at a distance and to connect doctors with each other. These services, mainly including online medical visits, virtual consultations, and remote monitoring and diagnosis, developed rapidly during the COVID-19 pandemic period [3-6]. As proved by former studies, telemedicine or virtual care has many advantages, such as decreased exposure risk to COVID-19 and other infectious diseases, ensuring the sustainability of medical services during quarantine; increased accessibility of medical services; lower costs for patients on both the money and time spent on the way to clinic; lower costs for medical institutes on patient flow management; relatively increased patient compliance, especially in chronic disease management; and early detection of subclinical changes [7-13]. Based on these advantages, telemedicine has been accepted, and both patients and providers are satisfied; it was even promoted by some policies as the pandemic proceeded [1,14-16].

Telemedicine in China developed early since the first remote consultation case in the mid-1980s, and the “Opinions on Promoting Telemedicine Services in Medical Institutions” policy issued in 2014 by the Chinese government accelerated its development [17.] In 2017, telemedicine platforms had been established in 22 of 34 provincial administrative regions in China that covered 13,000 medical institutions, which indicated that a telemedicine service system had been formed already [17,18]. When the COVID-19 pandemic exploded, this telemedicine system was rapidly put into practice. Virtual consultations were performed, live-stream platforms were used to offer health education to patients and the general public, virtual care bills were covered by the national health insurance, and medication-delivering systems were formed as the pandemic proceeded [2,19]. At some places, experts were gathered to offer preliminary screening for community residents with symptoms [20]. In Henan Province, the local telemedicine platform connected 147 COVID-19–designated hospitals, which offered prevention, diagnosis, and treatment of COVID-19 using modules such as real-time monitors, intractable case transfer, and remote guidance [21]. Implementation of telemedicine systems was on an unprecedented scale and pace.

In January 2023, the management of COVID-19 in China was changed from class A to class B, which means no longer isolating patients with COVID-19, no longer identifying close contacts, no longer designating high- and low-risk areas, and adjusting the testing strategy to voluntary testing. However, the pressure on the health care system rapidly increased in a short period after prevention measures were relaxed, which has led to different requirements for the application of telemedicine. Post–COVID-19, it is still uncertain how telemedicine would integrate in the health care system for a long time [22].

As a result, in this study, we aimed to compare the frequency of offering telemedicine services before the COVID-19, during the pandemic explosion, and after China reopened post–COVID-19 and to find out what kind of providers would like to offer more telemedicine services, even after pandemic prevention was relaxed.

## Methods

### Study Setting and Data Collection

The data for this study were taken from the 2023 Chinese National Survey of Telemedicine Development in Hospitals, conducted from September to October 2023, which used online questionnaires. The Telemedicine Informationization Professional Committee of China (TIPC) and the National Telemedicine Center of China (NTCC) were in charge of data collection for the survey.

A multistage sampling method was used to recruit respondents. Initially, 11 provinces were strategically selected from across eastern, central, and western regions for the survey, considering prior collaborative experiences. Subsequently, a TIPC member was designated to manage the survey operations in each province. They were tasked with overseeing hospital coordination and selection. Following receipt of consent from the chosen hospitals, a dedicated staff member was appointed in each facility by the TIPC members to handle the recruitment of participants, all of whom had undergone training by 3 experienced researchers from the NTCC. Ultimately, electronic questionnaires, accompanied by quick response (QR) codes and detailed instructions, were distributed to health care providers engaged in telemedicine services by the staff member in each hospital. Participation was voluntary, allowing participants to withdraw anytime. Data anonymity and confidentiality were guaranteed, and no monetary compensation was offered. Researchers from the NTCC periodically reviewed the data collected during the survey, offered prompt feedback, and carried out centralized data cleansing to ensure data integrity and effectiveness.

At the end of the survey, 996 questionnaires were received from all TIPC members. After excluding questionnaires with repeated information, incomplete responses, inconsistency, and other problems (eg, some providers reported having worked in telemedicine longer than their overall working years), there were 831 (83.43%) valid questionnaires left. Data used in this study were from questionnaires filled by health care providers who had ever practiced telemedicine and has answered the question “How frequently did you offer telemedicine services before/during/after the COVID-19 pandemic explosion?” As a result, only 428 (51.5%) questionnaires were included.

The questionnaires included the following demographic items:

- Age, sex, professional title, education, and working experience, especially in offering telemedicine services recorded by year
- Hospital information, such as province, level of the hospital, and which department the provider was from
- The provider’s feelings about validity and reliability (scoring from 1 to 10)
- Whether the provider engaged in telemedicine through a hospital self-operated platform or a platform run by a third party, which mainly refers to companies such as Chunyu Doctor
- The provider’s feelings about whether the time spent in offering telemedicine services is longer or shorter or not different compared to in-person visits and other offline medical services
- The provider’s feelings about how easy it is to acquire patients’ tests and examine results during the telemedicine service procedure
- The provider’s feelings about the frequency of offering telemedicine services before the COVID-19 pandemic outbreak, during the pandemic explosion and pandemic prevention, and after pandemic prevention control was relaxed

Professional titles included junior, intermediate, and senior. Education recorded doctor, master’s, and bachelor’s degrees, as well as education below the bachelor level. The province was then classified as the eastern, central, or western region of China. Hospital levels were categorized as tertiary or nontertiary. Departments of providers were sorted as clinic and nonclinic departments. How easy it was to acquire test results online and the frequency of telemedicine at 3 time points were scored on a 5-point Likert scale as “always,” “often,” “occasionally,” “rarely,” and “never.”

### Ethical Considerations

This study was conducted using an online survey method, collecting data that were limited to demographic information, such as age and gender, with no collection of sensitive or individually identifiable biological information. All procedures were approved by the Medical Ethics Committee (2020-KY-0379-002) of the First Affiliated Hospital of Zhengzhou University. Informed consent was obtained from all individual participants involved in the study. For secondary analyses of research data, we confirmed that the original informed consent or Institutional Review Board (IRB) approval included provisions allowing for secondary analysis without additional consent from the participants.

In this study, we prioritized the privacy and confidentiality of participants. The online survey was designed to collect only nonsensitive demographic information, such as age and gender, without any personal identifiers that could compromise the privacy of the respondents. All responses were collected anonymously, ensuring that no individual could be identified using the data.

Participants were not offered any form of compensation for their participation. No images of individual participants or users were included in the manuscript or multimedia appendices that would allow for identification.

### Outcomes

The general frequency of offering telemedicine services was calculated at each time point, and the difference in the frequency between the 3 groups was considered the primary outcome of this study. As the frequency was an ordered categorical variable, it was reassigned a score of 1-5 at each time point. A change in the frequency of offering telemedicine services as the pandemic progressed was calculated, via subtraction, as the difference in the frequencies between the 3 time points for each participant, and the change was categorized as an increase or a decrease, depending on whether the result of the subtraction was >0 or <0. Thus, binary variables showing whether the frequency increased from before the pandemic outbreak to during its explosion, increased from during the explosion to after controls were relaxed, and from before the outbreak to after controls were relaxed were calculated as secondary outcomes of this study.

### Statistical Analysis

Age was presented as the mean (SD). Other continuous variables were presented as the median (IQR) as their distribution was not symmetric. Categorical variables were described as counts and percentages. Chi-square tests were performed to compare differences in the frequency of offering telemedicine services between all groups. Single-variate and multiple regression analyses were used to explore correlations between the increase in frequency and other questionnaire responses. Stratified analysis was also performed in using the multiple regression model in different groups. A 2-sided *P* value of <05 was considered statistically significant in all analyses. Statistical analysis was performed using R version 4.3.1 (R Foundation for Statistical Computing). Figures demonstrating intergroup distributions and advantages and disadvantages were created in Microsoft Excel.

## Results

### Participant Details

A total of 428 health care providers (n=199, 46.5%, males and n=229, 53.5%, females) filled in the questionnaires and were included in this study. The general characteristics of participants and their responses to the questionnaires are summarized in Table 1. The participants’ mean age was 38.6 (SD 8.51) years. Over half were bachelors (n=257, 60.05%), with a median of 12 (IQR 7-20) years of working experience and 4 (IQR 2-6) years working in telemedicine. The majority were from tertiary hospitals (n=229, 53.5%) and located in central (n=218, 50.93%) and western (n=151, 35.28%) parts of China. Most providers were engaged in telemedicine through platforms operated by their own institutes (n=317, 74.07%), while the rest were engaged through third-party platforms usually operated by internet companies, such as Chunyu Doctor. Participants reported that test and examination results could be generally acquired (n=125, 29.21%, always; n=191, 44.63%, often) during the telemedicine procedure. Most participants believed that the reliability and validity of telemedicine are high, with a median score of 9 (IQR 8-10) and 9 (IQR 7.75-10), respectively, on a scale of 0-10. Regarding the complexity of the diagnosis procedure, 200 (46.73%) participants indicated online virtual visits are quicker than in-person outpatient visits, 99 (23.13%) participants reported the length of virtual medical service is even longer than an offline visit, and the rest reported that the time spent in the 2 methods is not different.

**Table 1:** General characteristics and responses of participants (N=428).

Characteristics			Value
Age (years), mean (SD)			38.6 (8.51)
**Sex, n (%)**			
	Female	229 (53.50)	
**Professional title, n (%)**			
	Junior	123 (28.74)	
	Intermediate	146 (34.11)	
	Senior	159 (37.15)	
**Education, n (%)**			
	Doctor	23 (5.37)	
	Master’s degree	88 (20.56)	
	Bachelor’s degree	257 (60.05)	
	Below bachelor’s degree	60 (14.02)	
Working years, median (IQR)			12 (7-20)
Telemedicine working years, median (IQR)			4 (2-6)
**Department, n (%)**			
	Clinic	370 (86.45)	
	Nonclinic	58 (13.55)	
**Region, n (%)**			
	Eastern	59 (13.79)	
	Central	218 (50.93)	
	Western	151 (35.28)	
**Type of hospital, n (%)**			
	Tertiary	229 (53.50)	
	Nontertiary	199 (46.50)	
**Telemedicine platform, n (%)**			
	Self-operated	317 (74.07)	
	Third party	111 (25.93)	
**Time spent in visits, n (%)**			
	Shorter online visits	200 (46.73)	
	Not different	129 (30.14)	
	Longer online visits	99 (23.13)	
**Acquire test results online, n (%)**			
	Never	6 (1.40)	
	Rarely	28 (6.54)	
	Occasionally	78 (18.22)	
	Often	191 (44.63)	
	Always	125 (29.21)	
Validity of telemedicine, median (IQR)			9 (8-10)
Reliability of telemedicine, median (IQR)			9 (7.75-10)

### Outcomes

According to the providers’ responses, the frequency of providing telemedicine services, including online visits, generally increased as the pandemic proceeded through the 3 stages: before, during, and after the COVID-19 explosion. As shown in Table 2, approximately 198 (46.26%) providers always or often offered telemedicine before the pandemic outbreak, 249 (58.17%) during the pandemic explosion, and 268 (62.62%) after the pandemic explosion. The differences between these groups, calculated using the chi-squared test, indicated that generally the frequency of providing telemedicine services increased since the pandemic exploded and stayed at similar levels after the explosion (Table 3).

**Table 2:** Frequency of offering telemedicine before, during, and after the COVID-19 pandemic.

Grade	Before the pandemic outbreak, n (%)	During the pandemic explosion, n (%)	After prevention measures were relaxed, n (%)
Never	29 (6.78)	11 (2.57)	9 (2.10)
Rarely	76 (17.76)	60 (14.02)	46 (10.75)
Occasionally	125 (29.21)	108 (25.23)	105 (24.53)
Often	159 (37.15)	196 (45.79)	214 (50.00)
Always	39 (9.11)	53 (12.38)	54 (12.62)

**Table 3:** Chi-square test results of differences between the frequencies of offering telemedicine.

Comparison between groups	*χ*^2^ (*df*)	*P* value
Before vs during the pandemic	17.21 (4)	.002^a^
During vs after the pandemic	2.89 (4)	.57
Before vs after the pandemic	30.17 (4)	<.001^a^

^a^*P*<.05.

Regression models were performed in single and multiple variate analyses, and whether offering telemedicine services increased during the pandemic explosion versus before the pandemic outbreak, after the pandemic explosion versus during the explosion, and after the pandemic explosion versus before the pandemic outbreak was considered a dependent variable. As demonstrated in Table 4, age, working experience, telemedicine working experience, and providers’ report of the validity of telemedicine were positively correlated or negatively correlated with an increase in the frequency of offering telemedicine services as the pandemic explosion proceeded, with significant correlation but a coeffective value close to 1. The providers’ professional titles were significantly correlated with the frequency increase in the single-variate model: they had an increased frequency compared to those with junior titles. Participants from western regions compared to central regions and from nontertiary hospitals compared to tertiary hospitals were less likely to increase telemedicine services as the pandemic exploded. Compared to participants who felt the time spent between online and in-person visits was not different, those who felt online services take longer (during vs before: odds ratio [OR] 4.27, 95% CI 2.30-8.15; after vs before: OR 3.22, 95% CI 1.79-5.92) or shorter (during vs before: OR 2.09, 95% CI 1.19-3.80; after vs before: OR 2.15, 95% CI 1.28-3.72) increased telemedicine services.

**Table 4:** Single-variate analysis of telemedicine service frequency increase during versus before the COVID-19 pandemic and after versus before the pandemic.

Characteristics			During vs before the pandemic				After vs before the pandemic		
			OR^a^ (95% CI)		*P* value		OR (95% CI)		*P* value
Age			1.04 (1.01-1.07)		.004^b^		1.01 (0.98-1.03)		.60
**Sex**									
	Male	0.91 (0.59-1.40)		.70		0.82 (0.54-1.24)		.40	
**Professional title**									
	Intermediate	1.97 (1.05-3.79)		.04		1.17 (0.68-2.04)		.60	
	Senior	3.99 (2.22-7.47)		<.001^b^		1.98 (1.19-3.36)		.01^b^	
**Education**									
	Bachelor’s degree	2.04 (0.99-4.63)		.07		1.38 (0.73-2.74)		.30	
	Master’s degree	2.93 (1.31-7.09)		.01^b^		1.97 (0.96-4.22)		.07	
	Doctor	2.48 (0.78-7.76)		.12		2.11 (0.74-5.93)		.20	
	Working years	1.04 (1.01-1.06)		.003^b^		1.01 (0.99-1.03)		.50	
	Telemedicine working years	0.99 (0.94-1.04)		.70		0.95 (0.89-0.99)		.04	
**Department**									
	Nonclinic	1.28 (0.69-2.32)		.40		1.57 (0.88-2.77)		.12	
**Region**									
	Eastern	1.69 (0.92-3.05)		.09		1.18 (0.64-2.13)		.60	
	Western	0.54 (0.32-0.88)		.02^b^		0.64 (0.40-1.02)		.07	
**Type of hospital**									
	Nontertiary	0.45 (0.29-0.70)		<.001^b^		0.63 (0.41-0.95)		.03^b^	
**Telemedicine platform**									
	Third party	0.74 (0.44-1.22)		.30		0.61 (0.36-0.99)		.05	
**Time spent in visits**									
	Longer online visits	4.27 (2.30-8.15)		<.001^b^		3.22 (1.79-5.92)		<.001^b^	
	Shorter online visits	2.09 (1.19-3.80)		.01^b^		2.15 (1.28-3.72)		.005^b^	
**Acquire test result online**									
	Often	1.02 (0.61-1.70)		.90		0.93 (0.57-1.52)		.80	
	Occasionally	1.03 (0.54-1.94)		.90		1.28 (0.70-2.33)		.40	
	Rarely	1.12 (0.43-2.70)		.80		1.08 (0.43-2.56)		.90	
	Never	0.56 (0.03-3.63)		.60		1.14 (0.15-6.13)		.90	
Validity of telemedicine			0.85 (0.75-0.97)		.02^b^		0.92 (0.81-1.05)		.20
Reliability of telemedicine			0.82 (0.72-0.94)		.70		0.89 (0.79-1.01)		.08

^a^OR: odds ratio.

^b^*P*<.05.

In the multivariate analysis, age, professional title, education, years working in telemedicine, region, type of hospital, platform provider engaged in telemedicine, time spent in online versus in-person service, and validity of telemedicine were included as independent variables in regression models. According to variance inflation factor test results shown in Table S1 in Multimedia Appendix 1, working years (during vs before: 10.85; after vs during: 9.99; after vs before: 10.86) and reliability of telemedicine (during vs before: 6.9; after vs during: 6.72; after vs before: 6.85) were not adjusted in the multivariate regression model. As shown in Table 5, providers with senior professional titles compared to junior titles, who were engaged in telemedicine through third party–operated platforms compared to hospital-run platforms, and who felt online visits are shorter or longer than in-person visits had significant coeffective values. In Tables S2 and S3 in Multimedia Appendix 2, single and multiple regression models involving frequency increases after versus during the pandemic explosion are reported, and no significant correlations were found.

**Table 5:** Multivariate analysis of telemedicine service frequency increase during versus before the COVID-19 pandemic and after versus before the pandemic.

Characteristics			During vs before the pandemic					After vs before the pandemic		
			OR^a^ (95% CI)^a^		*P* value		OR (95% CI)			*P* value
Age			0.99 (0.95-1.03)		.70		0.97 (0.93-1.01)			.20
**Professional title**										
	Intermediate	1.79 (0.84-3.95)		.14		1.38 (0.70-2.75)			.40	
	Senior	4.38 (1.72-11.6)		.002^b^		3.47 (1.46-8.49)			.005^b^	
**Education**										
	Bachelor’s degree	0.71 (0.29-1.85)		.50		0.74 (0.34-1.68)			.50	
	Master’s degree	0.69 (0.24-2.09)		.50		0.84 (0.32-2.22)			.70	
	Doctor	0.39 (0.09-1.59)		.20		0.83 (0.23-2.96)			.80	
Telemedicine working years			0.97 (0.92-1.03)		.40		0.93 (0.87-1.0)			.04^b^
**Region**										
	Eastern	1.82 (0.94-3.51)		.07		1.19 (0.61-2.26)			.60	
	Western	0.6 (0.32-1.10)		.10		0.62 (0.35-1.08)			.10	
**Type of hospital**										
	Nontertiary	0.79 (0.44-1.41)		.40		1.07 (0.62-1.86)			.80	
**Telemedicine platform**										
	Third party	0.57 (0.32-1.00)		.05		0.51 (0.29-0.86)			.02^b^	
**Time spent in visits**										
	Longer online visits	3.87 (1.95-7.89)		<.001^b^		3.14 (1.64-6.11)			<.001^b^	
	Shorter online visits	2.04 (1.11-3.87)		.02^b^		2.27 (1.31-4.07)			.004^b^	
Validity of telemedicine			0.96 (0.83-1.12)		.60		1 (0.86-1.16)			.90

^a^OR: odds ratio.

^b^*P*<.05.

Stratified analysis was performed. The distribution of the online service length between 2 types of platforms (Table S4 in Multimedia Appendix 3) showed that over half of the providers on self-operated platforms (160/317, 50.47%) felt online visits are shorter, while on third party–operated platforms, only 40 (36.04%) of 111 providers felt online visits are shorter. The difference in distribution between the 2 types of platforms was significant. Thus, the correlation of whether online service takes longer and the frequency increase in telemedicine might be different between the 2 types of platforms. As a result, providers were grouped by whether they chose to offer telemedicine on platforms operated by their own hospitals or on third party platforms.

As demonstrated in Table 6, multiple regression analysis was applied, and the variables age, professional title, education, years working in telemedicine, region of hospital, type of hospital, and validity of telemedicine reported by providers were adjusted as covariables in the regression model in each group. In the self-operated platform group, compared to providers who felt the time spent in online and in-person visits is not different, those who felt online visits are longer were significantly more likely to increase offering telemedicine services when COVID-19 exploded (OR 3.84, 95% CI 1.73-8.83) and when pandemic prevention controls were relaxed (OR 3.40, 95% CI 1.61-7.34) compared to before the pandemic. The differences in the telemedicine offer frequency increase for providers who felt online service time is shorter than in-person visits were not significant. In the third party–operated platform group, the increase in telemedicine offer frequency was significant among providers who felt online visits are shorter than in-person visits both when the pandemic exploded (OR 8.16, 95% CI 1.39-68.3) and when prevention measures were relaxed (OR 5.70, 95% CI 1.22-33.6) compared to before the pandemic, while the differences for providers who felt online visits are longer were not significant.

**Table 6:** Stratified analysis of the relationship between visit length and frequency increase^a^.

Platform type, visit length, and frequency change duration		OR^b^ (95% CI)	*P* value
**Self-operated: longer online visits**			
	During vs before the pandemic	3.84 (1.73-8.83)	.001^c^
	After vs before the pandemic	3.40 (1.61-7.34)	.001^c^
**Self-operated: shorter online visits**			
	During vs before the pandemic	1.61 (0.81-3.29)	.20
	After vs before the pandemic	1.78 (0.96-3.41)	.07
**Third party:** **longer online visits**			
	During vs before the pandemic	6.51 (1.05-54.7)	.06
	After vs before the pandemic	1.87 (0.32-11.6)	.50
**Third party:** **: shorter online visits**			
	During vs before the pandemic	8.16 (1.39-68.3)	.03^c^
	After vs before the pandemic	5.70 (1.22-33.6)	.04^c^

^a^Adjusted for age, professional title, education, years working in telemedicine, region, type of hospital, and validity of telemedicine.

^b^OR: odds ratio.

^c^*P*<.05.

## Discussion

### Principal Findings

Based on a national survey of telemedicine providers, this study explored the change in the frequency of offering telemedicine services at the time before COVID-19 exploded, during the pandemic explosion, and after prevention measures were relaxed; analyzed the correlation between other factors and the frequency change; and located health care providers who would like to offer more telemedicine services in China even after the pandemic is over.

In general, our study found that the frequency of health care providers offering telemedicine increased both during the pandemic and after prevention measures were relaxed compared to before the pandemic. Specifically, we found that providers’ feelings about the online service length compared to in-person visits were correlated with the telemedicine offer frequency increase as the pandemic proceeded. Among providers offering telemedicine through platforms run by their own hospitals, those who felt online visits take longer were more likely to increase offering telemedicine. Among providers engaged through third-party platforms, those who felt online visits are shorter were more likely to increase offering telemedicine.

### Comparison With Prior Work

Studies worldwide have already proved that telemedicine technologies were helpful tools during the COVID-19 explosion and were generally welcomed after the pandemic was over [1,3,9,23,24]. Considering the condition changed in China, it was significant that the telemedicine offer frequency was still high after pandemic controls were relaxed [22].

In our study, the difference in the telemedicine frequency change was not significant between different types of hospitals but was significant between the 2 types of platforms. As Chinese telemedicine-promoting policies started early, there were various ways of offering telemedicine services at the time the pandemic exploded [17]. In May 2019, there were 158 telemedicine platforms known as “internet hospitals” operated by local public hospitals around the nation and video-based platforms, such as the Huawei telepresence conference system [18]. Private companies, such as Chunyu Doctor (founded in 2011) and Ping An Health (founded in 2015), established and began operating their teleconsulting and diagnosis platforms even earlier. Some physicians said they would still contact patients through phone calls, text messages, or instant messaging software, such as WeChat, but a few responded that they would offer telemedicine services or only consultations through these ways. As a result, common tools, such as WhatsApp, telephone, or email, were not included as a way of offering telemedicine in this study. The difference in frequency between the 2 kinds of platforms was not the same as the platforms or tools used in former studies [25,26].

The 2 types of platforms in this study included providers engaging in telemedicine publicly or privately. As all our participants were from public hospitals, the self-operated platforms were similar to those run by public hospitals. However, the third-party platforms were mainly run by private companies. Former studies on cognition and telemedicine services between public and private providers are not consistent. Galle et al [27] and Cordioli et al [28] suggested that providers in the private sector or private clinics have a higher frequency of offering telemedicine services. Scheffer et al [29] claimed physicians working in both public and private sectors are the most frequent users. Elhadi et al [30] indicated there are no differences in attitude, cognition, knowledge, or skills in providers between work in public hospitals, private hospitals, or both. Mazouri-Karker et al [31] reported that differences between public and private providers are only their preference in tools. In our results, public providers reported that a higher frequency increase after pandemic controls were relaxed might be because public hospitals, especially tertiary public hospitals in China, usually have a better reputation, whereas private third-party telemedicine platforms or physicians who work a “part-time job” on these platforms can hardly acquire patient trust.

We also found that some providers feel that online visits take longer, others believe that online visits are shorter, while still others feel that they take the same time as in-person visits. This is consistent with Ly’s [32] results that some physicians report telemedicine increases their efficiency and lowers the workload, while other physicians report telemedicine takes more time and increases their workload because of having to learn new techniques and because communication on-screen is difficult. Although some researchers believe that one of the advantages of adopting telemedicine during COVID-19 was that it could save time and increase efficiency, it actually saved patients’ traffic-related costs on both time and money and increased efficiency by reducing unnecessary visits through pretriage online [24,30,33]; however, we are still uncertain about whether adopting telemedicine could have saved the providers’ time. Naamani et al [34] suggested that telemedicine could save providers’ time because of pretriage online. Shaarani et al [26] proved that the average time spent in in-person visits reported by former studies is longer than 15 minutes, while providers in their survey reported a shorter average online visit time. Silver et al [35] found out that providers who spent an average visiting time longer than 14 minutes would prefer offering visits in person instead of virtually. Ramsey et al [36] indicated that providers would have to spend more time on documentation, which made the procedure more complicated and online visits took longer.

Providers in our study who reported that online visits are shorter than in-person visits have a good reason to increase the frequency of offering telemedicine services as these have higher efficiency. Providers who reported that online visits are longer than in-person visits, compared to those who believed there is no difference between the 2 service types, would also like to increase the telemedicine usage frequency. This might be because telemedicine has other advantages as well, such as saving patients’ costs, even though it would increase the time spent by providers. As Ramsey et al [36] suggested, the other benefits for clinicians, patients, and quality of care could motivate health care professionals to offer more telemedicine services. In addition, in China, the average outpatient visiting time is much shorter, especially in tertiary hospitals [37]. This indicates that even though online visits take longer, they might still be shorter than average in-person visits in other countries. Providers who feel online visits take more time are not against offering telemedicine according to Silver et al [35], as also shown by our results of stratified analysis. Providers offering telemedicine services through their own hospitals’ platforms might be more responsible and attach importance to their reputation. However, providers using third-party platforms might pay more attention to efficiency. As a result, on self-operated platforms, the increase in the telemedicine offer frequency by providers who felt online visits take longer was significant, while on third-party platforms, the increase in the telemedicine offer frequency by providers who felt online visits are shorter was significant.

### Limitations

Our study showed that the length of online visits is correlated with the frequency change in offering telemedicine by providers, and the frequency increased for both longer and shorter online visits. However, our questionnaire was not designed to acquire the average number of minutes the providers spent in online and in-person visits. Further studies could focus on this to obtain a more detailed and convincing result. The results from this study were all from a cross-sectional survey, though it was nationwide. Thus, the frequencies of offering telemedicine at the 3 time points and the frequency change data were all obtained from providers' self-reports, which may have led to self-report and selection bias. It would be more accurate after regional online prescription–examining platforms in China are established and we can acquire data on them.

Moreover, some questions in this study were rather subjective. As the questionnaire used a 5-point Likert scale, questions regarding the frequency of offering telemedicine was set with 5 levels from “never” to “always,” without specifying the exact time or duration for each level. “What platform do you mainly use to offer telemedicine services?” was set as a single-choice question, which was less suitable for more complicated situations, such as providers using both types of platforms.

### Conclusion

The frequency of telemedicine services offered by health care providers has increased since the COVID-19 pandemic exploded and was still high after pandemic prevention controls were relaxed in China. Differences in providers’ professional titles, types of platforms, and length of online visits compared to in-person visits are correlated with the increase in frequency. In using hospital-run platforms, providers with longer online visits are more likely to offer telemedicine services, while on third-party platforms, providers with shorter online visits would like to increase telemedicine services.

## Appendix

Multimedia Appendix 1 Variance inflation factor test.

Multimedia Appendix 2 Single and multiple regression model for frequency increase between after and during groups.

Multimedia Appendix 3 Group difference in online visit lengths between platforms.

## Abbreviations

NTCC: National Telemedicine Center of China
OR: odds ratio
TIPC: Telemedicine Informationization Professional Committee of China

## Data Availability

The datasets generated and analyzed during this study are not publicly available because they are questionnaire data obtained from a nationwide survey initiated by the Telemedicine Informationization Professional Committee of China, but they are available from the corresponding author upon reasonable request.

## References

[1] Bokolo Jr. A (2020). Use of telemedicine and virtual care for remote treatment in response to COVID-19 pandemic. J Med Syst.

[2] Webster P (2020). Virtual health care in the era of COVID-19. Lancet.

[3] Bokolo AJ (2021). Exploring the adoption of telemedicine and virtual software for care of outpatients during and after COVID-19 pandemic. Ir J Med Sci.

[4] Silva RSD, Schmtiz CAA, Harzheim E, Molina-Bastos CG, Oliveira EBD, Roman R, Umpierre RN, Gonçalves MR (2021). The role of telehealth in the covid-19 pandemic: a Brazilian experience. Cien Saude Colet.

[5] Combi C, Pozzani G, Pozzi G (2016). Telemedicine for developing countries. a survey and some design issues. Appl Clin Inform.

[6] Schwamm LH, Estrada J, Erskine A, Licurse A (2020). Virtual care: new models of caring for our patients and workforce. Lancet Digit Health.

[7] Maroongroge S, De B, Woodhouse KD, Bassett RL, Lee P, Bloom ES, Smith GL, Frank SJ, Li J, Perkins G, Das P, Koong AC, Smith BD, Wang C (2023). Physician perspectives on telemedicine in radiation oncology. Adv Radiat Oncol.

[8] Liu J, Liu S, Zheng T, Bi Y (2021). Physicians' perspectives of telemedicine during the COVID-19 pandemic in China: qualitative survey study. JMIR Med Inform.

[9] Cerda IH, Therond A, Moreau S, Studer K, Donjow AR, Crowther JE, Mazzolenis ME, Lang M, Tolba R, Gilligan C, Ashina S, Kaye AD, Yong RJ, Schatman ME, Robinson CL (2024). Telehealth and virtual reality technologies in chronic pain management: a narrative review. Curr Pain Headache Rep.

[10] Bellicini MG, D'Altilia FP, Gussago C, Adamo M, Lombardi CM, Tomasoni D, Inciardi RM, Metra M, Pagnesi M (2023). Telemedicine for the treatment of heart failure: new opportunities after COVID-19. J Cardiovasc Med (Hagerstown).

[11] Sumner L, Schmidt H, Minden S, Falkenberg N, Sun L, McBurney R, Loud S, Wallin M (2023). Use of telemedicine among people with multiple sclerosis before and during the COVID-19 pandemic. Telemed J E Health.

[12] Saleem MK, Sattar K, Ejaz KF, Rehman MU, Saleem H, Khursheed S, Akbar A, Ahmed J, Tariq M, Jadoon SK, Saleem Khan M, Tasneem S, Khandker SS, Kundu S, Alvi S (2024). Use of telemedicine to tackle health problems in South Asia during the COVID-19 era and beyond: a systematic review. Ann Med Surg (Lond).

[13] Mahajan V, Singh T, Azad C (2020). Using telemedicine during the COVID-19 pandemic. Indian Pediatr.

[14] Aldekhyyel RN, Alshuaibi F, Alsaaid O, Bin Moammar F, Alanazy T, Namshah A, Altassan K, Aldekhyyel R, Jamal A (2024). Exploring behavioral intention to use telemedicine services post COVID-19: a cross sectional study in Saudi Arabia. Front Public Health.

[15] Johnsen TM, Norberg BL, Kristiansen E, Zanaboni P, Austad B, Krogh FH, Getz L (2021). Suitability of video consultations during the COVID-19 pandemic lockdown: cross-sectional survey among Norwegian general practitioners. J Med Internet Res.

[16] Kim J, Cai ZR, Chen ML, Onyeka S, Ko JM, Linos E (2024). Telehealth utilization and associations in the United States during the third year of the COVID-19 pandemic: population-based survey study in 2022. JMIR Public Health Surveill.

[17] Zhang W, He D, Wang G, Zhu C, Evans R (2022). Analyzing national telemedicine policies in China from the perspective of policy instrument (1997-2020). Int J Med Inform.

[18] Cui F, Ma Q, He X, Zhai Y, Zhao J, Chen B, Sun D, Shi J, Cao M, Wang Z (2020). Implementation and application of telemedicine in China: cross-sectional study. JMIR Mhealth Uhealth.

[19] Chen R, Jiang Q (2022). Evolution of telemedicine in China during COVID-19 pandemic: from 2020 to 2022. J Public Health Policy.

[20] Song X, Liu X, Wang C (2020). The role of telemedicine during the COVID-19 epidemic in China-experience from Shandong Province. Crit Care.

[21] Gao J, Ma Q, Sun D, Yang Y, Ren M, Wang L, Fan C, Fan Z, Cao M, Zhao J (2023). Telemedicine in the battle with 2019 novel coronavirus disease (COVID-19) in Henan Province, China: a narrative study. Telemed J E Health.

[22] Ge J (2023). The COVID-19 pandemic in China: from dynamic zero-COVID to current policy. Herz.

[23] Thomas EE, Haydon HM, Mehrotra A, Caffery LJ, Snoswell CL, Banbury A, Smith AC (2022). Building on the momentum: sustaining telehealth beyond COVID-19. J Telemed Telecare.

[24] Gareev I, Gallyametdinov A, Beylerli O, Valitov E, Alyshov A, Pavlov V, Izmailov A, Zhao S (2021). The opportunities and challenges of telemedicine during COVID-19 pandemic. Front Biosci (Elite Ed).

[25] Nies S, Patel S, Shafer M, Longman L, Sharif I, Pina P (2021). Understanding physicians' preferences for telemedicine during the COVID-19 pandemic: cross-sectional study. JMIR Form Res.

[26] Shaarani I, Ghanem A, Jounblat M, Jounblat H, Mansour R, Taleb R (2022). Utilization of telemedicine by the Lebanese physicians during time of pandemic. Telemed J E Health.

[27] Galle A, Semaan A, Huysmans E, Audet C, Asefa A, Delvaux T, Afolabi BB, El Ayadi AM, Benova L (2021). A double-edged sword-telemedicine for maternal care during COVID-19: findings from a global mixed-methods study of healthcare providers. BMJ Glob Health.

[28] Cordioli E, Giavina-Bianchi M, Pedrotti CHS, Podgaec S (2023). Brazilian Medical Survey on Telemedicine since the onset of COVID-19. Einstein (Sao Paulo).

[29] Scheffer M, Cassenote A, de Britto E Alves MTSS, Russo G (2022). The multiple uses of telemedicine during the pandemic: the evidence from a cross-sectional survey of medical doctors in Brazil. Global Health.

[30] Elhadi M, Elhadi A, Bouhuwaish A, Bin Alshiteewi F, Elmabrouk A, Alsuyihili A, Alhashimi A, Khel S, Elgherwi A, Alsoufi A, Albakoush A, Abdulmalik A (2021). Telemedicine awareness, knowledge, attitude, and skills of health care workers in a low-resource country during the COVID-19 pandemic: cross-sectional study. J Med Internet Res.

[31] Mazouri-Karker S, Lüchinger R, Braillard O, Bajwa N, Achab S, Hudelson P, Dominicé Dao M, Junod Perron N (2023). Perceptions of and preferences for telemedicine use since the early stages of the COVID-19 pandemic: cross-sectional survey of patients and physicians. JMIR Hum Factors.

[32] Ly BA, Labonté R, Bourgeault IL, Niang MN (2017). The individual and contextual determinants of the use of telemedicine: a descriptive study of the perceptions of Senegal's physicians and telemedicine projects managers. PLoS One.

[33] Grossman Z, Chodick G, Reingold SM, Chapnick G, Ashkenazi S (2020). The future of telemedicine visits after COVID-19: perceptions of primary care pediatricians. Isr J Health Policy Res.

[34] El Naamani K, Abbas R, Mukhtar S, El Fadel O, Sathe A, Kazan AS, El Hajjar R, Sioutas GS, Tjoumakaris SI, Menachem Maimonides Bhaskar S, Herial NA, Gooch MR, Rosenwasser RH, Jabbour P (2022). Telemedicine during and post-COVID 19: the insights of neurosurgery patients and physicians. J Clin Neurosci.

[35] Silver SL, Lewis MN, Ledford CJW (2021). A stepwise transition to telemedicine in response to COVID-19. J Am Board Fam Med.

[36] Ramsey A, Mustafa SS, Portnoy JM (2022). Patient and clinician attitudes toward telemedicine for allergy and immunology. J Allergy Clin Immunol Pract.

[37] Ren W, Sun L, Tarimo CS, Li Q, Wu J (2021). The situation and influencing factors of outpatient satisfaction in large hospitals: evidence from Henan Province, China. BMC Health Serv Res.

