# Supplementary material for: Correlation Between the Online Visiting Time and Frequency Increase in Telemedicine Services Offered by Health Care Providers Before, During, and After the COVID-19 Pandemic in China: Cross-Sectional Study
Source: J Med Internet Res. 2025 Feb 26;27:e65092. doi: 10.2196/65092 (PMC11904373; doi:10.2196/65092)
Supplement: Multimedia Appendix 1 [file jmir_v27i1e65092_app1.docx]

**Table S1.** Variance inflation factor test.

| **Variables** | **VIF** | **Df** | **VIF** | **Df** | **VIF** | **Df** |
| --- | --- | --- | --- | --- | --- | --- |
|  | During vs Before | | After vs During | | After vs Before | |
| Region | 1.44 | 2 | 1.49 | 2 | 1.48 | 2 |
| Type of hospitals | 1.53 | 1 | 1.58 | 1 | 1.61 | 1 |
| Telemedicine platform | 1.18 | 1 | 1.16 | 1 | 1.17 | 1 |
| Time spent of visiting | 1.29 | 2 | 1.24 | 2 | 1.27 | 2 |
| Sex | 1.11 | 1 | 1.11 | 1 | 1.11 | 1 |
| Age | 11.62^a^ | 1 | 10.85^a^ | 1 | 11.76^a^ | 1 |
| Professional title | 2.84 | 2 | 3.15 | 2 | 3.16 | 2 |
| Education | 2.18 | 3 | 2.22 | 3 | 2.29 | 3 |
| Working years | 10.85^a^ | 1 | 9.99^a^ | 1 | 10.86^a^ | 1 |
| Telemedicine working years | 1.31 | 1 | 1.36 | 1 | 1.35 | 1 |
| Department | 1.17 | 1 | 1.13 | 1 | 1.15 | 1 |
| Acquire test result online | 1.47 | 4 | 1.45 | 4 | 1.47 | 4 |
| Validity of telemedicine | 6.83^a^ | 1 | 6.6^a^ | 1 | 6.77^a^ | 1 |
| Reliability of telemedicine | 6.9^a^ | 1 | 6.72^a^ | 1 | 6.85^a^ | 1 |
| ^a^VIF > 5 | | | | | | |
