# Supplementary material for: Correlation Between the Online Visiting Time and Frequency Increase in Telemedicine Services Offered by Health Care Providers Before, During, and After the COVID-19 Pandemic in China: Cross-Sectional Study
Source: J Med Internet Res. 2025 Feb 26;27:e65092. doi: 10.2196/65092 (PMC11904373; doi:10.2196/65092)
Supplement: Multimedia Appendix 2 [file jmir_v27i1e65092_app2.docx]

**Table S2.** Single variate analysis between group after vs during.

| **Characteristic** | **OR (95% CI)**^a^ | ***P*** |
| --- | --- | --- |
| Age | 0.97(0.94, 1.00) | .047 |
| Sex | | |
| Male | 0.8(0.49, 1.30) | .4 |
| Professional title | | |
| Intermediate | 1.07(0.59, 1.96) | .8 |
| Senior | 0.84(0.46, 1.56) | .6 |
| Education | | |
| Bachelor | 1.4(0.67, 3.21) | .4 |
| Master | 1.16(0.48, 2.96) | .7 |
| Doctor | 2(0.60, 6.40) | .2 |
| Working years | 0.98(0.95, 1.00) | .086 |
| Telemedicine working years | 0.94(0.88, 1.00) | .073 |
| Department | | |
| Non-clinic | 1.28(0.63, 2.45) | .5 |
| Region | | |
| Eastern | 0.93(0.43, 1.89) | .9 |
| Western | 0.89(0.52, 1.50) | .7 |
| Type of hospitals | | |
| Non-tertiary | 0.9(0.55, 1.47) | .7 |
| Telemedicine platform | | |
| 3rd party | 0.78(0.43, 1.36) | .4 |
| Time spent of visiting | | |
| Online visit longer | 2.06(1.07, 4.04) | .032^b^ |
| Online visit shorter | 1.27(0.70, 2.37) | .4 |
| Acquire test result online | | |
| Often | 1.11(0.62, 2.04) | .7 |
| Occasionally | 1.49(0.73, 3.01) | .3 |
| Rarely | 1.35(0.45, 3.58) | .6 |
| Never | 0.99(0.05, 6.57) | .9 |
| Validity of telemedicine | 1.02(0.88, 1.19) | .8 |
| Reliability of telemedicine | 0.98(0.84, 1.14) | .7 |
| ^a^OR = Odds Ratio, CI = Confidence Interval. ^b^*P* < .05 . | | |

**Table S3.** Multivariate analysis between group after vs during.

| **Characteristic** | **OR (95% CI)**^a^ | ***P*** |
| --- | --- | --- |
| Age | 0.96(0.91, 1.01) | .091 |
| Professional title |  |  |
| Intermediate | 1.18(0.55, 2.56) | .7 |
| Senior | 1.34(0.50, 3.71) | .6 |
| Education |  |  |
| Bachelor | 1.08(0.43, 2.87) | .9 |
| Master | 0.86(0.27, 2.80) | .8 |
| Doctor | 1.85(0.43, 7.70) | .4 |
| Telemedicine working years | 0.97(0.89, 1.04) | .4 |
| Region |  |  |
| Eastern | 1.21(0.53, 2.58) | .6 |
| Western | 1.12(0.58, 2.12) | .7 |
| Type of hospitals |  |  |
| Non-tertiary | 0.94(0.50, 1.78) | .9 |
| Telemedicine platform |  |  |
| 3rd party | 0.75(0.39, 1.37) | .4 |
| Time spent of visiting |  |  |
| Online visit longer | 2.09(1.01, 4.45) | .051 |
| Online visit shorter | 1.29(0.68, 2.55) | .4 |
| Validity of telemedicine | 1.08(0.91, 1.30) | .4 |
| ^a^OR = Odds Ratio, CI = Confidence Interval. |  |  |
