# Supplementary material for: Correlation Between the Online Visiting Time and Frequency Increase in Telemedicine Services Offered by Health Care Providers Before, During, and After the COVID-19 Pandemic in China: Cross-Sectional Study
Source: J Med Internet Res. 2025 Feb 26;27:e65092. doi: 10.2196/65092 (PMC11904373; doi:10.2196/65092)
Supplement: Multimedia Appendix 3 [file jmir_v27i1e65092_app3.docx]

**Table S4.** Group difference of online visit length between platforms.

| Grade | Groups | | P value* |
| --- | --- | --- | --- |
|  | Self_operated | Third_party |  |
| indifference | 93 ( 29.34 %) | 36 ( 32.43 %) | 0.014 |
| Online_visit_longer | 64 ( 20.19 %) | 35 ( 31.53 %) |  |
| Online_visit_shorter | 160 ( 50.47 %) | 40 ( 36.04 %) |  |
| *: Chi-square test were performed to estimate group difference | | | |
